# Genome Sequence of *Burkholderia plantarii* ZJ171, a Tropolone-Producing Bacterial Pathogen Responsible for Rice Seedling Blight

**DOI:** 10.1128/genomeA.01318-16

**Published:** 2016-12-08

**Authors:** Yuan Qian, Haruna Matsumoto, Wenzhuo Li, Guonian Zhu, Yasuyuki Hashidoko, Yang Hu, Mengcen Wang

**Affiliations:** a Institute of Pesticide and Environmental Toxicology, Zhejiang University, Hangzhou, China; b Research Faculty of Agriculture, Hokkaido University, Sapporo, Japan; c Department of Forest Mycology and Plant Pathology, Swedish University of Agricultural Science, Uppsala, Sweden

## Abstract

*Burkholderia plantarii* is the causal agent of rice seedling blight. Here, we report the draft genome sequence of *B. plantarii*, which contains 8,020,831 bp, with a G+C content of 68.66% and a predicted 7,688 coding sequences. The annotated genome sequence will provide further insight into its pathogenicity.

## GENOME ANNOUNCEMENT

*Burkholderia plantarii* is a rice-pathogenic bacterium causing seedling blight by the production of tropolone, a phytotoxin, as the virulence factor (1, 2). Rice seedlings showed typical symptoms, such as chlorosis, stunting, and root growth inhibition (3), when infested by *B. plantarii* or treated with tropolone only (4, 5). This disease was first reported in a rice nursery in Chiba Prefecture, Japan, in 1985 and was found to be widespread in other major rice-growing regions of Asia, including China (6). Particularly during recent years, the greenhouse has been adopted as an important measure for transplantation and production of rice seedlings in Asia, which fostered outbreaks of seedling blight and caused severe damages to rice cultivation (6). In this report, the genome sequence of the *B. plantarii* strain ZJ171 was determined, assembled, and annotated.

*B. plantarii* ZJ171 (16S rRNA gene under accession no. LC020026) was isolated from a rice paddy in Zhejiang Province, China (29°48.467′N, 120°17.867′E). The whole genome of *B. plantarii* ZJ171 was sequenced by Illumina HiSeq 2500 paired-end sequencing technology at Biomarker (Beijing, China). The reads were assembled using Velvet (7), and gene annotation was carried out using GLIMMER for contigs equal or longer than 1,000 bp (8). The assembled genome consisted of 150 contigs, 8,020,831 bp (G+C content of 68.66%), and an *N*_50_ of 98,282 bp. It comprised 7,688 coding sequences of genes and 58 predicted RNA genes (46 tRNA genes, 1 rRNA gene, and 11 microRNA genes).

Analysis of the genome of *B. plantarii* ZJ171 revealed critical genes involved in biofilm formation, tropolone production, and the regulatory, biosynthetic, and secretory systems related to tropolone production. Also, quorum sensing (QS) is known to regulate *B. plantarii* virulence (2). Previously, a pair of *N*-acyl homoserine lactone (AHL)-QS genes, *pla-plaR*, were found to be involved in activation of tropolone production (2). In the assembled genome of *B. plantarii* ZJ171, 2 additional paired AHL-QS genes and 21 orphan AHLs regulator-coding genes without the AHL synthase-coding genes (LuxR family transcriptional regulators) were found, indicating the more sophisticated regulatory patterns for the diverse responses to interspecies communication with the host plant and various competitors. Interestingly, it was first noticed that the gene coding for enoyl coenzyme A (CoA) hydratase (*rpfF*) was existed in *B. plantarii* ZJ171, which functions as the key component of the BDSF (*cis*-2-dodecenoic acid) signaling pathway controlling *Burkholderia cepacia* complex (Bcc) virulence (9, 10), suggesting the possible involvement of the *Burkholderia* diffusible signal factor (BDSF) pathway in the regulation of *B. plantarii* virulence*.* Similar to *Burkholderia glumae*, genes coding for virulence-related enzymes, such as lipase LipA (11) and LysR-type transcriptional activator (12), have also been detected in *B. plantarii* ZJ171.

### Accession number(s).

This whole-genome shotgun project has been deposited at DDBJ/ENA/GenBank under the accession no. MKGK00000000. The version described in this paper is version MKGK01000000. The BioProject designation for this project is PRJNA323430. The BioSample accession no. is SAMN05178893.
